# PoEMs edit breast cancer outcome

**DOI:** 10.18632/aging.102870

**Published:** 2020-02-26

**Authors:** Pawel Bieniasz-Krzywiec, Massimiliano Mazzone

**Affiliations:** 1 Laboratory of Tumor Inflammation and Angiogenesis, Center for Cancer Biology, VIB, Leuven B3000, Belgium; 2 Laboratory of Tumor Inflammation and Angiogenesis, Center for Cancer Biology, Department of Oncology, KU Leuven, Leuven B3000, Belgium

**Keywords:** Podoplanin, tumor-associated macrophages, galectin 8, lymphangiogenesis, breast cancer metastasis

Cancer occurs when cells refuse to die and keep multiplying while concealing themselves from the adaptive immune system. Remarkably, the innate immune infiltrate (e.g. macrophages, neutrophils and mast cells) often supports cancer progression, especially at later stages of tumor development. In this regard, myeloid-derived innate cells are greatly responsible for tumor promoting-inflammation and constitute the major source of (lymph)angiogenic factors, thus creating new routes of cancer escape and mediating resistance to anti-angiogenic therapies [1]. In mammary malignancies, tumor-associated macrophages (TAMs) represent the most abundant leukocyte population as they may account for over 50% of the tumor mass. Notably, high TAM infiltration in breast cancer is associated with active lymphangiogenesis, lymph node (LN) involvement and distant organ metastasis [2]. Nevertheless, the crosstalk between TAMs and lymphatics has not been well understood thus far, leaving the precise molecular mechanisms behind macrophage-induced lymphatic dissemination yet to be unraveled.

Recently, we have identified a subset of TAMs characterized by the expression of Podoplanin (PDPN) – a glycoprotein implicated in regulating cell motility and adhesion [3]. Up to date, PDPN has been studied mainly in the context of platelet aggregation (as a CLEC2 ligand) and as a marker of the lymphatic endothelium (distinguishing lymphatics from blood vessels). Interestingly, PDPN is often upregulated in inflammation-related conditions, including cancer, and its properties are generally pro-tumoral (e.g. through supporting cancer cell migration, epithelial-mesenchymal transition and T cell exhaustion) [4]. In our work, we assessed the expression of PDPN in murine breast tumor-infiltrating immune cells and found that it was almost exclusively expressed by TAMs. In various orthotopic breast cancer models, Podoplanin-expressing macrophages (PoEMs) constituted 30% of all TAMs and were not found outside of the tumors. Importantly, PoEMs localized in the close proximity to tumor lymphatics where they stimulated lymphangiogenesis – the growth of new lymphatic vessels from pre-existing conduits. Consistent with several reports that breast cancer cells spread predominantly via the lymphatic route [5], the presence of PoEMs in the perilymphatic niche correlated with LN and lung metastasis in our murine models.

Based on these observations, we further investigated why PoEMs were found in the lymphatic space and by what means their presence aided lymphangiogenesis. In a series of Transwell migration assays we identified Galectin 8 (GAL8) as a soluble factor released by lymphatic endothelial cells (LECs) that attracted PoEMs but not Podoplanin-negative macrophages (non-PoEMs). GAL8 is a glycan-binding protein often classified as an extracellular matrix (ECM) component with properties similar to fibronectin. At large, GAL8 acts as a molecular anchor regulating cell motility and adhesion, as its binding triggers integrin-mediated signaling cascades resulting in cytoskeleton rearrangement and directional cell movement [6]. In line, we found that PDPN and GAL8 interacted on macrophage surface and that this interaction unleashed the clustering and activation of β1 integrin in PoEMs. The latter was required for the chemotactic attraction and adhesion of PoEMs to lymphatic vessel walls, confirming the previously described importance of β1 signaling during macrophage migration [7]. *In vivo*, the deletion of PDPN in macrophages or the deletion of GAL8 in lymphatic vessels exerted very similar effects, namely impaired TAM-LV interactions, abridged tumor lymphangiogenesis and consequently diminished organ metastasis. The deletion of both proteins at the same time had no synergistic effects, confirming that PDPN in macrophages and GAL8 in lymphatics participate in the same molecular process which facilitates lymphangiogenesis in breast cancer.

**Figure 1:**
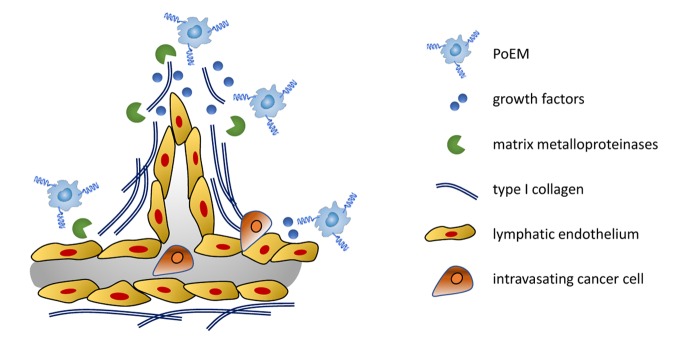
**In breast cancer, PoEMs migrate towards the lymphatic endothelium.** In this niche, they stimulate lymphangiogenesis by means of matrix remodeling resulting in the accessibility of growth factors. Moreover, Collagen I synthetized by PoEMs constitutes a scaffold for growing lymphatics. Directly and indirectly, matrix remodeling by PoEMs also fuels cancer cell lymphoinvasion.

In order to unveil the functional differences between PoEMs and non-PoEMs we sorted those populations from orthotopic 4T1 tumors and analyzed their transcriptomes. We found that PoEMs significantly upregulated genes related to extracellular matrix (ECM) remodeling, both in terms of matrix deposition (collagen subunits) and degradation (matrix metalloproteinases, MMPs). Unsurprisingly, ECM remodeling is of paramount importance during lymphangiogenesis, as LVs stay in direct contact with the matrix due to the incompletion of their basement membrane. Most commonly, lymphatic capillaries lay on type I collagen, whose various subunits were highly upregulated in PoEMs. Furthermore, sprouting lymphatic tip cells migrate towards growth factors, and those are liberated from the matrix due to the enzymatic activity of MMPs. Of note, ECM formation and degradation, as well as matrix stiffness, mechanically modulate the ability of cancer cells to migrate and invade LVs. Following, we confirmed the enhanced ability of PoEMs to deposit and digest various matrix components in several *in vitro* and in *vivo* assays, summarized in Figure 1.

Finally, we translated those findings to human breast cancer. First, we found that PoEMs represented 30% of the overall TAM population also in this context, and that they were able to migrate towards lymphatic endothelium-derived GAL8, mirroring our observations in mice. Then, we assessed the localization of PoEMs in tumor samples from a unique cohort of patients with bilateral tumors, in which one tumor was LN-positive (indicating LN involvement) and the other tumor was LN-negative. Remarkably, LN-positive tumors were characterized by a significantly higher frequency of PoEMs in the perilymphatic space as compared to LN-negative tumors. Moreover, elevated numbers of perilymphatic PoEMs in those samples correlated with higher rate of distant organ metastasis.

On the whole, our findings highlight an emerging concept that properties of macrophages are inherently related to specific niches they reside in. In recent years, a plenty of research has focused on TIE2-positve macrophages proximal to blood vessels (where they promote pro-angiogenic programs) [8]. On the other hand, our study describes a subset of TAMs marked by the expression of PDPN and uniquely associated with tumor lymphatics. We found that the presence of PoEMs in the lymphatic niche fosters lymphangiogenesis and aids cancer dissemination. Consequently, PDPN, PoEMs and GAL8 stem as potential prognostic biomarkers and therapeutic targets in breast cancer.
